# Investigating Potential Applications of the Fish Anti-Microbial Peptide Pleurocidin: A Systematic Review

**DOI:** 10.3390/ph14070687

**Published:** 2021-07-17

**Authors:** Katelyn A. M. McMillan, Melanie R. Power Coombs

**Affiliations:** 1 Biology Department, Acadia University, Wolfville, NS B4P 2R6, Canada; 143301m@acadiau.ca; 2 Department of Pathology, Dalhousie University, Halifax, NS B3H 4H7, Canada

**Keywords:** cancer, anti-microbial, peptide, pleurocidin, resistance

## Abstract

The anti-microbial peptide (AMP) pleurocidin is found in winter flounder (*Pseudopleuronectes americanus*), an Atlantic flounder species. There is promising evidence for clinical, aquaculture, and veterinary applications of pleurocidin. This review provides an overview of the current literature available on pleurocidin to guide future research directions. By fully elucidating pleurocidin’s mechanism of action and developing novel treatments against pathogenic microbes, populations of flatfish and humans can be protected. This review consulted publications from PubMed and Environment Complete with search terms such as “pleurocidin”, “winter flounder”, and “antimicrobial”. The fish immune system includes AMPs as a component of the innate immune system. Pleurocidin, one of these AMPs, has been found to be effective against various Gram-positive and Gram-negative bacteria. More investigations are required to determine pleurocidin’s suitability as a treatment against antibiotic-resistant pathogens. There is promising evidence for pleurocidin as a novel anti-cancer therapy. The peptide has been found to display potent anti-cancer effects against human cancer cells. Research efforts focused on pleurocidin may result in novel treatment strategies against antibiotic-resistant bacteria and cancer. More research is required to determine if the peptide is a suitable candidate to be developed into a novel anti-microbial treatment. Some of the microbes susceptible to the peptide are also pathogens of fish, suggesting its suitability as a therapeutic treatment for fish species.

## 1. Introduction

Anti-microbial peptides (AMPs) are a widely studied group of molecules that are approximately 12–50 amino acids in length [1]. They are naturally occurring components of the innate immune system in many different organisms and show great promise in being developed into novel antibiotic treatments against microbes. AMPs’ value as potential novel treatments have been reviewed previously, specifically on optimization and peptide specificity, and stability of peptides, highlighting common mechanisms of action [2]. There is promising research outlining the potential of AMPs to be developed into therapies, but there are many factors to consider when developing a novel therapy. As such, extensive research into the properties of these peptides and their interactions *in vivo* are required to determine if the AMP has clinical potential. This review examines the nature of pleurocidins, which are AMPs from Atlantic flatfish. A broad overview of the fish immune system will also be discussed, as well as a brief examination of common fish pathogens. Considering the current literature, there are multiple potential applications for pleurocidin. This review highlights clinical and aquacultural applications.

Winter flounder is an Atlantic marine species of flatfish that is found in more shallow waters along the eastern coast of North America [3]. Adults move from deeper waters into estuaries to spawn eggs in late winter to early spring, which is unique when compared to other Atlantic fish species [3]. Winter flounder consumes a broad diet mostly consisting of annelids and amphipods and is described as an opportunistic feeder [3]. A component of the innate immune system in this fish is AMPs, including pleurocidins, the focus of this study.

Pleurocidin is an amphipathic α-helical peptide [4]. This peptide is derived from the skin mucosa and intestinal secretions of winter flounder and displays a broad range of anti-microbial activity against Gram-positive and Gram-negative bacterial pathogens [4,5]. Pleurocidin attacks membranes by the toroidal pore and carpet model, as well as inhibit internal metabolic processes, all of which lead to cell death [6]. This review will also discuss the pathogenicity of common bacterial fish pathogens, *Vibrio anguillarum* and *Aeromonas salmonicida,* as well as the viral hemorrhagic septicemia virus (VHSV). Pleurocidin’s effect against these microbes will be discussed, as well as its effect on viruses and cancers. This review describes how fish are able to prevent infection in a few different ways, with AMPs being at the forefront of the innate immune response. Future steps for developing pleurocidin into a potential treatment against disease in both fish and humans will be explored. Extensive literature on this peptide is lacking; thus, further research on the suitability of pleurocidin for these human and aquatic applications is warranted. This review aims to be a guide for future researchers to identify a research focus.

## 2. Materials and Methods

Current literature was reviewed on pleurocidin, examining its natural role in winter flounder and potential preclinical relevance. The databases PubMed and Environment Complete were chosen for the study to ensure relevant papers were consulted on the clinical and aquacultural aspects of pleurocidin. The inclusion criteria for consulted studies were the key terms “pleurocidin” and “winter flounder”. An additional search with the terms “pleurocidin” and “*Pseudopleuronectes americanus*” revealed no new articles. However, some relevant papers on pleurocidin’s effect on human cancer cells and fish pathogens were included despite not mentioning “winter flounder”, to ensure a thorough account on the current literature of the anti-cancer potential of pleurocidin.

To provide sufficient context to disease in fish, other search terms were used. These terms included “fish pathogens”, “winter flounder pathogens”, and “fish immune system”. Relevant studies on the fish immune system and fish pathogens were also reviewed. The specific pathogens *V. anguillarum*, *A. salmonicida*, and the viral pathogen VHSV were investigated as they were identified as common fish pathogens.

Papers relevant to the preclinical applications of pleurocidin were found using the search terms “pleurocidin, cancer” and “winter flounder, cancer”. Papers describing the general mechanism of action of AMPs were found using the search terms “anti-microbial peptide, mechanism of action”, “AMP, mechanism of action”, and “AMP mode of attack”.

**Figure 1:**
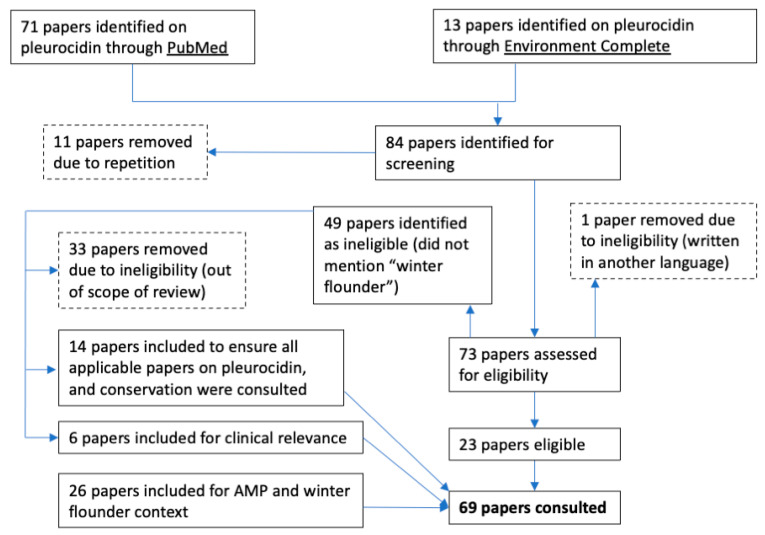
PubMed and Environment Complete diagram of papers included in the review of pleurocidin.

All available papers from the databases PubMed and Environment Complete were screened for eligibility (Figure 1). No date restrictions or filters were used in the search. Papers excluded from the review are outlined with dashed lines (Figure 1). In total, 43 papers were consulted specifically on pleurocidin. The additional searches resulted in a total of 69 papers consulted.

**Figure 2:**
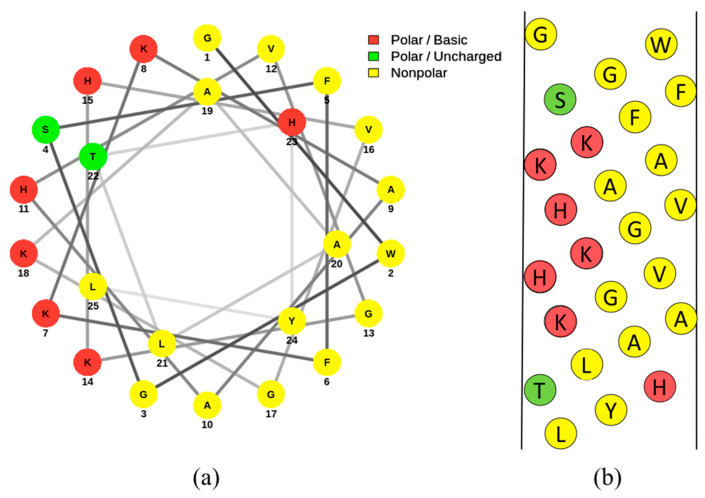
Helical wheel (**a**) and net diagrams (**b**) of pleurocidin [4]. Amino acids are indicated by their single-letter abbreviations and are categorized according to the following colors: red (polar basic), green (polar uncharged), and yellow (nonpolar). Both the wheel and net diagrams demonstrate the division of hydrophobic and hydrophilic components of the peptide. A slightly acidic pH is assumed, as the histidine residues are labelled as polar. The helical wheel diagram was generated using the online program NetWheels [35] and the net diagram was made using Microsoft PowerPoint.

**Figure 3:**
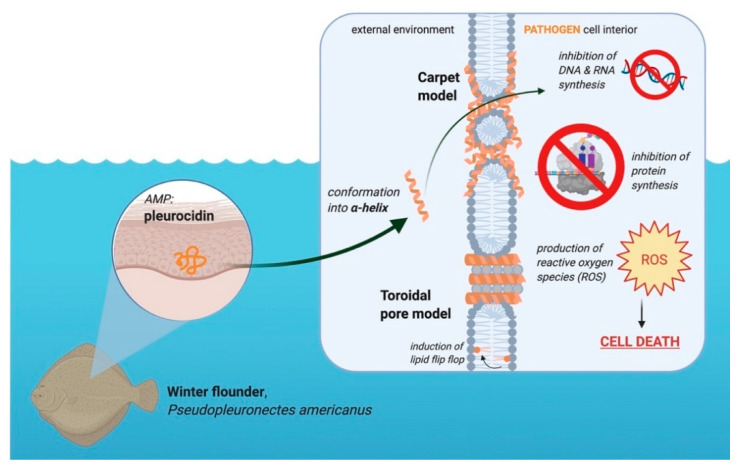
Role of the AMP pleurocidin in the winter flounder in the defense against pathogens. This peptide is located in the granular cells of winter flounder skin. Upon interacting with a negatively charged membrane, the peptide conforms into an α-helix. Made in © BioRender—biorender.com accessed on 31 May 2021.

The software used to make the figures in this paper included NetWheels, Microsoft PowerPoint, and © BioRender. NetWheels was used to make Figure 2a. PowerPoint was used to make Figure 1 and Figure 2b. Lastly, © BioRender was used to make the graphical abstract and Figure 3.

## 3. Results

### 3.1. Fish Immunity

Fish have innate defense mechanisms consisting of pre-existing and inducible components that allow for their protection. This group of vertebrates comprises about 40% of all vertebrate species, and thus an effective immune system is essential to ensure species are not lost to disease and that diversity is maintained [7]. Due to the close contact of fish with pathogens in the environment, it is crucial that the immune response is fast acting and broad spectrum [7]. The adaptive immune system in fish involves a longer response time and the production of T lymphocytes, B cells, and antibodies [8]. Studies have found gut-associated T lymphocytes in teleost fish with activity against a variety of fish viruses [8]. Despite the presence of an adaptive immune response in fish, the production of antibodies may take weeks; therefore, the innate immune system is heavily relied on by fish species to prevent infection [9]. Key components of the innate immune system in fish include the complement system, lysozymes, and AMPs [7,10]. The complement system in fish helps to combat pathogens by recruiting inflammatory mediators and disrupting the cell membrane of the pathogen [9]. Lysozymes are enzymes that break down the peptidoglycan in bacterial cell walls, and are therefore important in combatting bacterial infections [9,11]. Since the innate immune response is much more fast-acting than the temperature-dependent adaptive response, it is not a surprise that the action of lysozymes and complement in fish is more potent than that of higher vertebrates [9,12]. AMPs are also crucial components of the fish innate immune system. They are typically found on the mucosal skin surface of fish and are effective at preventing infection by a variety of microbes [13,14]. In combination, these innate and adaptive systems allow fish to effectively fight off infection. That being said, further research into the immune response of fish, specifically the role of AMPs, can help us better understand how to prevent disease in fish.

### 3.2. Fish Pathogens

Fish are an important food source; therefore, the aquaculture industry is prevalent in many cultures. Like other cultivated animals, fish are susceptible to disease and populations of farmed fish suffer from infection, most notably from bacterial diseases [10]. Populations of fish in more polluted bodies of water are more susceptible to disease, with higher volumes of antibodies being detected in fish from water polluted with sewage waste [15]. Bacterial pathogens such as *V. anguillarum, A. salmonicida,* and the viral pathogen VHSV are a few of many fish pathogens [9,16,17]. *Vibrio anguillarum* is a more extensively studied bacterial pathogen that afflicts winter flounder and other species in both marine and brackish water [18]. *Vibrio anguillarum* are Gram-negative curved-rod bacteria that cause the formation of ulcers, gross lesions, and necrosis of the fins in adult winter flounder [17,18]. Inflammation of the dermis occurs with low numbers of lymphocytes observed in the inflamed tissue [17]. Myolysis, the breakdown of muscle tissue, is also observed in muscles below areas of ulcer formation in infected fish [17]. Once the pathogen gains access to the internal organs, the intestines can fill with liquid and become distended [18]. Additionally, infection by this bacterium can occur following exposure to as little as 670 *V. anguillarum* cells, suggesting that this disease poses a significant threat to winter flounder populations [17]. The bacterium likely enters by mouth or through the skin of fish [18]. Since AMPs are abundant on fish skin, studying their effect on pathogens such as *V. anguillarum* will help us better understand how to prevent infection in fish. Indeed, some studies have already found that mucus isolates from winter flounder are effective at preventing the growth of *V. anguillarum* [14].

*Aeromonas salmonicida* is another bacterial species that is particularly pathogenic towards fish. It causes furunculosis, a disease that results in systemic infection [19]. This bacterium tends to infect fish species such as salmon and trout, but has also been found in summer and winter flounder [15,16]. Pleurocidin is effective against both *V. anguillarum* and *A. salmonicida*, suggesting AMPs play a role in fighting infection [16]. Antibodies to these bacteria have also been found in flounder, providing evidence that the adaptive immune system plays a role in the prevention of disease as well [15]. That being said, winter flounder had about 1/10 as many antibodies in sera as other fish species; therefore, innate components of the immune system might be more effective at preventing infection by these bacterial species [15].

VHSV is a rhabdovirus that causes hemorrhaging, lethargy, and erratic swimming in infected finfish, which include flounder species [20]. VHSV is a problem for aquaculture worldwide because the disease is highly fatal for infected fish [20]. These bacterial and viral diseases of fish have devastating effects on the aquaculture industry due to losses of adult fish [9,20]. Efforts have been made to develop a vaccine to VHSV for both freshwater and marine fish; however, a vaccine is not yet commercially available [21]. By continually studying the fish immune response to these pathogens we can better understand how to prevent widespread infection of these pathogens. In addition to vaccines, studying the roles that AMPs play in the immune response may lead to a more effective and cost-efficient treatment.

### 3.3. Pleurocidin

Pleurocidin is an α-helical peptide, composed of 25 amino acid residues, which is localized in the skin and intestine secretions of winter flounder [4,14,22]. The pleurocidin peptide was initially isolated in 1997 [4]. Later studies were able to isolate up to 20 other pleurocidin peptides from various species of Atlantic flatfish and named the peptides “NRC”, numbering them from 01 to 20 [23].

**Figure 4:**
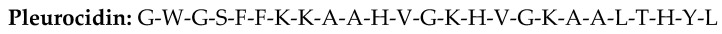
Amino acid sequence of initial pleurocidin peptide NRC-04 [4].

Pleurocidin plays an important role in the innate immune system of this fish species. Other fish peptides showing homology to pleurocidin are found in species such as striped bass [24]. Studies have shown that most of the expression of pleurocidin occurs in the skin and that there are clusters of pleurocidin genes on the winter flounder’s genome [22,25]. The amino acid sequence of pleurocidin is shown in Figure 4. Pleurocidin-like peptides all have conserved flanking regions of amino acids, but the middle sequences of amino acids tend to differ [23]. These peptides have been found to display similar anti-microbial effects as the original pleurocidin peptide [23,26]. An image of the amino acid distribution of pleurocidin is shown in Figure 2 in a helical wheel and net diagram. Since this peptide has not been found in any other tissue of the flounder, pleurocidin is assumed to be skin-specific and involved in host defense in the digestive tract and on the skin surface [27]. Some studies have suggested the expression of pleurocidin is regulated in response to infection and inflammation [27]. This peptide originates primarily from the nonlamellar tissue of the gills, and is expressed in winter flounder as early as 13 days after hatching [25,28]. In this way, pleurocidin is a component of the flounder’s immune system and acts to kill any invading pathogens that come into contact with the flounder. This peptide shows homology with other classes of AMPs, specifically Anuran dermaseptins [29] and ceratotoxins isolated from the Mediterranean fruit fly [4,30]. Therefore, it is not surprising that pleurocidin demonstrates broad-spectrum anti-microbial activity against a wide range of Gram-positive and Gram-negative bacteria, including methicillin-resistant *Staphylococcus aureus* (MRSA) [4,5,31]. Included in this list of pathogens are the foodborne bacteria *Vibrio parahemolyticus*, *Escherichia coli* O157:H7, *Listeria monocytogenes*, *Saccharomyces cerevisiae*, and *Penicillium expansum* [31]. That being said, some bacteria are resistant to the anti-microbial activity of pleurocidin. Species of bacteria that are particularly resistant to pleurocidin include *Enterococcus faecalis*, a commensal microbe of the mammalian gastrointestinal tract [32]. On the other hand, *Vibrio* species are particularly susceptible to pleurocidin and other AMPs as compared to a wide variety of other Gram-positive and Gram-negative bacterial species [33,34]. This suggests the winter flounder has adapted an immune system that is more effective against the species of microbes that pose a significant threat.

Pleurocidin, when conformed to an amphipathic α-helix, is mainly hydrophobic on one face and hydrophilic on the other [4]. Figure 2a and Figure 2b demonstrate this amphipathic structure in a Schiffer–Edmundson wheel diagram and net diagram, respectively. This amphipathic property allows for pleurocidin to actively interact with the polar and nonpolar components of cell membranes. Studies that investigate the action of AMPs such as pleurocidin typically use model membranes composed of zwitterionic phosphatidylcholine (PC) or phosphatidylcholine/phosphatidylglycerol (PC/PG) membranes to elucidate the peptide interactions with various types of membrane bilayers [36,37]. Upon contact with anionic membranes, pleurocidin conforms to an α-helix [37]. Like other AMPs, the tryptophan residues on the peptide allow pleurocidin to anchor to the membrane surface [37]. The formation of this secondary structure occurs in the presence of charged molecules; pleurocidin does not conform to a helix in the presence of zwitterionic phospholipids [36,37]. The reason behind this is due to electrostatic repulsions between the NH_3_ groups of aromatic residues of the peptide and the positive charge of choline on the neutral phospholipids [37]. Studies have shown that adjusting the structure of pleurocidin can alter the helical content when exposed to a variety of membranes [38]. Needless to say, the ability of a peptide to conform to a helix and bind to a membrane is sensitive to small changes in the peptide structure. Once the peptide interacts with the lipid bilayer, pores are formed in the membrane. As described previously, AMPs interact with phospholipid bilayers in a few different ways [39,40,41]. Knowledge on pleurocidin’s mechanism of action is somewhat lacking; however, there are studies that have described the interaction with microbes [37].

### 3.4. Pleurocidin’s Mechanism of Action

Pleurocidin’s mechanism of action involves a combination of membrane permeation and metabolic inhibition (Figure 3). Pleurocidin either acts by the carpet or toroidal pore model, with some hypothesizing that the peptide permeates membranes using components of both models [37,42,43]. Whether or not pleurocidin acts by the toroidal pore or carpet model depends on the concentration of peptide compared to the lipid bilayer [37]. At higher peptide-to-lipid ratios, pleurocidin forms toroidal pores in cell membranes, whereas at lower peptide-to-lipid ratios the peptide permeates via the carpet mechanism [36,37,42]. Like other AMPs, pleurocidin is most active against negatively charged cell membranes as opposed to neutral membranes [43]. In addition to membrane disruption, pleurocidin inhibits metabolic processes, including inhibiting DNA synthesis and migration [5,6]. At the minimum inhibitory concentration (MIC), pleurocidin inhibits RNA and DNA synthesis in bacteria, effectively preventing the cells from producing proteins [6]. Pleurocidin also causes the upregulation of reactive oxygen species (ROS) in *Candida albicans*, which ultimately results in apoptosis [44]. Membrane permeation and metabolic inhibition make pleurocidin an effective AMP against a variety of microbes.

Like other AMPs, pleurocidin inserts into the lipid bilayer through the N-terminus residues, which penetrate deeper into the membrane than other amino acids in the sequence [37]. These residues at the N-terminus include aromatic amino acids, such as tryptophan and phenylalanine [37]. The N-terminus is important in maintaining the α-helical structure of pleurocidin [37]. Permeation of membranes by pleurocidin is also facilitated by the disruption of the acyl chains of the phospholipids, with anionic lipids being more susceptible to disorder [36,37]. The formation of these toroidal pores, in high amounts, may cause leakage of essential molecules from the affected cells and eventually cause cell death [6]. Hybrids of pleurocidin have also been found to cause lipid flip-flop without extensive permeabilization of the membrane [6]. High concentrations of peptide are required to cause this lipid flip-flop, though not as high as the concentrations needed to cause calcein leakage from liposomes treated with the peptide [6,36].

### 3.5. Optimization of Pleurocidin

Despite the effectiveness of pleurocidin, several studies have investigated ways of enhancing the anti-microbial properties of the peptide. These methods include substituting amino acids, using enantiomeric pleurocidin, forming peptide staples, and altering the pleurocidin gene. Following the substitution of the glycine residues on the N-terminal of the peptide with amino acids that increase α-helicity, the antibacterial activity of pleurocidin is hypothesized to increase [45]. This is because the glycine residues lower the stability of the α-helical structure, which is critical for proper membrane interactions [45]. Other amino acid substitutions have also been used to decrease the hemolytic activity of the peptide, which shows that the peptide is capable of being optimized for therapeutic use [46]. Similarly to substituting amino acids, the anti-microbial activity of pleurocidin has been amplified by altering the enantiomeric amino acids [47]. This study found that enantiomeric pleurocidin was more active against *Candida albicans*, a fungal pathogen, and was more resistant to degradation by proteases [47,48]. Forming pleurocidin molecules with staples, which are hydrocarbon linkages between amino acids, also helps to stabilize the structure of the peptide. One study used a design algorithm to form pleurocidin stapled AMPs that exhibited varying levels of stability and hemolytic abilities [49]. By using genetic technologies, one study produced non-amidated pleurocidin molecules, some of which were found to be more effective at killing microbes [50]. Pleurocidin has the ability to enhance the activity of conventional antibiotics, suggesting the peptide can be used to improve the efficacy of currently available treatments [51].

### 3.6. Potential Clinical Anti-Microbial and Anti-Cancer Applications

While pleurocidin is effective against bacterial cells, the peptide has very low hemolytic effects against human erythrocytes, which is likely due to the prevalence of cholesterol in the red blood cell membrane [37]. This characteristic suggests pleurocidin might be suitable as a therapeutic agent against both human pathogens and cancer, as the cholesterol abundance in these cell types is different from normal, healthy cells [52]. Pleurocidin kills human pathogens such as *Streptococcus mutans* [53] and human cancers such as breast cancer [32,48]. Pleurocidin is also effective against *Streptococcus sobrinus*, another Gram-negative species related to dental caries [54]. Pleurocidin is resistant to fluctuations in physiological concentrations of magnesium and calcium, suggesting the activity of the peptide would be stable in the human blood stream [27]. That being said, the presence of enzymes in whole blood might affect the stability of pleurocidin. Nevertheless, these observations suggest pleurocidin may be suitable as a novel antibiotic treatment. Due to the rising antibiotic resistance by pathogens such as MRSA, the need for new treatments against bacterial pathogens is paramount [55]. Steps have already been taken in integrating the pleurocidin gene into vectors in order to optimize the mass synthesis of the peptide for therapeutic use [56]. By continuing to study this peptide’s anti-microbial action, we will better understand pleurocidin’s potential as a novel antibiotic.

Investigations into the anti-cancer effects of pleurocidin have found that the peptides NRC-03 and NRC-07 from the pleurocidin family are effective at killing both multiple myeloma and breast carcinoma cells [57,58]. NRC-03 was more cytotoxic than NRC-07 against both cancer types [57,58]. Therefore, it is not surprising that NRC-03 has also been found to be highly toxic to human leukemia cells, with much lower levels of toxicity to normal human cells [59]. This peptide causes DNA fragmentation, as well as upregulating ROS, suggesting a metabolic anti-cancer mechanism in addition to membrane destabilization [57,58]. Interestingly, at lower doses the peptide is able to increase the susceptibility of cancer cells to current chemotherapies, suggesting its possible use as an adjuvant [58]. [D]-enantiomeric NRC-03 is more cytotoxic against breast cancer than NRC-03 and is non-hemolytic [48]. That being said, the enantiomeric peptide was more cytotoxic to other normal cells, which could be prevented by adjusting the amino acid sequence to reduce this toxicity [48]. Epinecidin-1, a peptide similar in structure to pleurocidin and isolated from grouper fish (*Epinephelus coioides*) is able to induce apoptosis and cause mitochondrial damage in populations of human leukemia cells [60]. These studies demonstrate the effectiveness of pleurocidin and pleurocidin-like peptides against cancer cells and their ability to be optimized for anti-cancer activity.

Despite the effectiveness of these peptides, some breast cancer variants have been formed that are resistant to pleurocidin peptides due to an altered cell membrane composition [61]. However, these peptide-resistant cancer cells are less efficient at forming tumors in immune-deficient mice, suggesting that despite the resistance to the anti-cancer peptides, these cancer cell variants are not very aggressive and pleurocidin could still be used as an adjuvant treatment [61]. Pleurocidin peptides have also been found to stimulate the human immune system by activating mast cells, a component of the innate immune system involved in allergy and inflammation [62]. This study determined that pleurocidin peptides, specifically NRC-04, are able to induce granulation of mast cells and the production of chemokines [62]. These studies show that pleurocidin has potential as a novel anti-cancer agent and possibly as a treatment to enhance the action of the immune system.

### 3.7. Conservational Applications of Pleurocidin

Populations of both wild and farmed fish are affected by pathogens all over the globe. AMPs are promising new tools to confer resistance in these fish populations against deleterious pathogens. Pleurocidins, may help protect fish populations as immune response modulators. Researchers have already developed effective vaccine strategies for fish against *Vibrio harveyi*, whereby the AMP and protein adjuvants are encased in polymers [63,64]. One study found pleurocidins to confer long-term immunity against *V. harveyi* in grouper by promoting lymphocyte proliferation and TNF-α [64]. Another study attempted to use a pleurocidin promoter to protect against VHSV, but found the promotor did not induce an effective response compared to other promotors [65]. *Vibrio parahaemolyticus* also poses a problem for farmed and wild fish populations [66]. One study determined that a specific group of membrane proteins of this bacterial species confer resistance to pleurocidin. Four inner membrane proteins and two outer membrane proteins related to protein efflux and ATP synthesis were upregulated in pleurocidin-resistant *V. parahaemolyticus* cells [66]. This provides a mechanism through which pleurocidin can be optimized to target or bypass these membrane components to ensure resistant strains of *Vibrio* are susceptible to treatment. Pleurocidin has also been found to be effective against the oyster parasite *Perkinsus marinus*, although not as effective as other AMPs [67].

## 4. Discussion

The broad spectrum of activity of AMPs suggests that these molecules can be applied in many different disciplines, including as novel antibiotic treatments. Following the initial study that isolated and characterized pleurocidin in 1997, the same researchers attempted to better our understanding of the expression of the pleurocidin gene and the peptide’s spectrum of activity [27]. The current literature on pleurocidin presents avenues for research into the potential therapeutic uses of pleurocidin in both humans and fish. One study also found that the peptide is effective against an oyster parasite. Global populations of fish are declining for many different reasons, including global warming, disease, and increased fishing [56,57]. By studying the potential for AMPs to render fish resistant to common pathogens, these populations can be protected from unnecessary degradation, essentially protecting the population and the community of organisms in aquatic ecosystems. On the other hand, pleurocidin also shows promise as a novel human therapy against cancer and bacterial pathogens. There are some interesting papers investigating the cytotoxic abilities of the AMP against breast and myeloma cancer [48,57,58]. While there is not enough evidence to conclude clinical potential, these observations warrant further investigation into pleurocidin as a preclinical candidate for future research. Similarly, the aquacultural applications of pleurocidin require further investigation.

The potential applications of this peptide depend on pleurocidin’s mechanism of action and compatibility *in vivo*. Pleurocidin has been found to be strongly cytotoxic to pathogenic Gram-negative species of bacteria, as well as other relevant pathogens in aquaculture and human cancers. The important findings on pleurocidin are outlined in Table 1. Limitations to this systematic review include the availability of current studies on the consulted databases PubMed and Environment Complete. Both databases were chosen to take into account the clinical and aquaculture aspects of the peptide pleurocidin, respectively. Despite these databases containing a broad range of publications, there were some studies that were omitted from the searches because they did not focus on potential clinical or conservational aspects of pleurocidin.

To provide context for the aquacultural applications of pleurocidin, this review outlined the effect of pleurocidin against three known fish pathogens, *Vibrio* species, *Aeromonas salmonicida*, and the viral pathogen VHSV. There may also be other fish pathogens not mentioned in this review that are more susceptible to pleurocidin, as an investigation of all fish pathogens is out of the scope of this review. There is not exhaustive literature on pleurocidin, which suggests that the peptide is relatively unknown and there is still much to elucidate in regard to pleurocidin’s suitability as a novel therapeutic treatment.

Future directions in research into pleurocidin include continued investigation of its mechanism of action against common bacterial pathogens. Due to the prevalence of *Vibrio* species as fish pathogens, and pleurocidin being seemingly more effective against Gram-negative species, research should focus on these fish pathogens. Research on the most virulent pathogens in Atlantic fish populations may confirm which pathogens should be targeted. In addition to fish pathogens, the effectiveness of pleurocidin against antibiotic-resistant strains of bacteria should be investigated. Due to increasing resistance to antibiotics in bacterial pathogens, developing novel therapies is a priority.

Efforts should also be made to investigate the peptide as a novel anti-cancer agent because there is evidence that the peptide is nonhemolytic and effective at killing some types of human cancer cells. Modifications to the original peptide may also be conducted in order to enhance pleurocidin’s anti-microbial activity. Studies have already determined that the action of this peptide can be enhanced with the use of these optimization methods. Oncolytic peptides have been designed and observed to cause mitochondria perturbation in melanoma cells, resulting in an inflammatory immune response and cancer cell death [68]. Pleurocidin should therefore be investigated for possible induction of immune responses to cancer in future studies on the preclinical potential of the peptide. A possible drawback to the use of pleurocidin as a treatment is the stability of the peptide inside the target organisms, therefore peptide optimization will be key. Through these efforts, a novel therapeutic treatment could be developed to treat both aquatic populations and human populations suffering from disease.

## 5. Conclusions

Pleurocidin is an effective AMP against a variety of Gram-positive and Gram-negative bacteria, in addition to killing cancer cells. This peptide attacks cell membranes by the carpet and toroidal pore model as well as blocking internal cell metabolism. These actions all result in cell death. There is promising evidence that pleurocidin has preclinical potential as a novel cancer treatment and as a treatment against fish pathogens. The potential of pleurocidin to induce inflammatory immune responses to cancer should also be explored. In light of the growing resistance of disease-causing bacteria to conventional antibiotics, more research on pleurocidin is required to determine if the peptide would be an effective novel antibiotic against antibiotic-resistant bacterial species. As most of the resistant species are Gram-negative [69], particular focus should be placed on determining pleurocidin’s suitability against these targets.

## Figures and Tables

**Table 1:** Important characteristics of pleurocidin relevant to the peptide’s potential human and aquaculture applications.

Potential Applications	Pleurocidin Characteristics	Reference(s)
Human	Low hemolysis	[37]
	Induction of DNA fragmentation in cancer cells	[57]
	Upregulation of ROS in cancer cells and *C. albicans*	[44,58]
	Induction of mast cell granulation	[62]
	Cytotoxic against breast cancer cells	[48,58]
	Cytotoxic against myeloma cells	[57]
	Cytotoxic against leukemia cells	[60,61]
	Cytotoxic against MRSA	[31]
	Cytotoxic against *S. mutans* and *S. sobrinus*	[32,53,54]
	Effective against foodborne pathogens *V. parahemolyticus*, *E. coli* O157:H7, *L. monocytogenes*, *S. cerevisiae*, and *P. expansum*	[31]
Aquaculture	Potential vaccine component against *V. harveyi*	[64]
	Effective against oyster *P. marinus*	[67]

## Data Availability

Data sharing not applicable.
